# Biochemistry and transcriptomic analyses of *Phthorimaea* *absoluta* (Lepidoptera: Gelechiidae) response to insecticides

**DOI:** 10.1038/s41598-024-58413-z

**Published:** 2024-04-04

**Authors:** Samantha W. Karanu, Inusa J. Ajene, Elijah K. Lelmen, Maureen A. Ong’onge, Komivi S. Akutse, Fathiya M. Khamis

**Affiliations:** 1 International Centre of Insect Physiology and Ecology, Nairobi, Kenya; 2 Department of Biochemistry, Egerton University, Egerton, Kenya; 3 Unit for Environmental Sciences and Management, North-West University, Potchefstroom, 2520 South Africa

**Keywords:** Biochemistry, Biological techniques, Molecular biology

## Abstract

*Phthorimaea* *absoluta* is an invasive solanaceous plant pest with highly devastating effects on tomato plant*.* Heavy reliance on insecticide use to tackle the pest has been linked to insecticide resistance selection in *P.* *absoluta* populations. To underline insights on *P.* *absoluta* insecticide resistance mechanisms to diamides and avermectins, we evaluated the transcriptomic profile of parental (field-collected) and F8 (lab-reared) populations. Furthermore, to screen for the presence of organophosphate and pyrethroid resistance, we assessed the gene expression levels of acetylcholinesterase (*ace1*) and *para*-type voltage-gated sodium channel (VGSG) genes in the F1 to F8 lab-reared progeny of diamide and avermectin exposed *P.* *absoluta* field-collected populations. The VGSG gene showed up-regulation in 12.5% and down-regulation in 87.5% of the screened populations, while *ace1* gene showed up-regulation in 37.5% and down-regulation in 62.5% of the screened populations. Gene ontology of the differentially expressed genes from both parental and eighth generations of diamide-sprayed *P.* *absoluta* populations revealed three genes involved in the metabolic detoxification of diamides in *P.* *absoluta*. Therefore, our study showed that the detoxification enzymes found could be responsible for *P.* *absoluta* diamide-based resistance, while behavioural resistance, which is stimulus-dependent, could be attributed to *P.* *absoluta* avermectin resistance.

## Introduction

The tomato leafminer, *Phthorimaea* *absoluta* (Meyrick) (Lepidoptera: Gelechiidae), is considered the most highly destructive tomato pest originating from Peru, South America^1^. The pest’s invasion of Spain in 2006 through agricultural trade, led to a widespread invasion across Afro-Eurasia, posing a danger to tomato production globally^2^. If left unmanaged, the pest can cause 80–100% yield losses on tomato production attributed to the larval leaf-mining activity and tomato fruit infestation. Insecticide use is the predominant control strategy for *P.* *absoluta* due to lack of effective sustainable alternative control strategies, and which consequently led to insecticide over-reliance and misuse to overcome the pest pressure^3^.

Although integrated pest management strategies such as biological, cultural, genetic and behavioural control methods have been implemented and pursued, chemical control still remains the most dominant and preferred *P.* *absoluta* management strategy^4^. However, successive introduction of chemical compounds with insecticide over-reliance led to insecticide resistance selection in *P.* *absoluta* populations based on the chemical compounds used^4^. Insecticide resistance poses a high threat of further pest invasion, tomato production yield losses and possible *P.* *absoluta* control failure^5^. To alleviate insecticide resistance in *P.* *absoluta* populations and the resultant problems, mechanisms involved in *P.* *absoluta* insecticide resistance should be exhaustively explored.

According to the Insecticide Resistance Action Committee (IRAC), insecticide resistance is defined as “a heritable change in the sensitivity of a pest population that is reflected in the repeated failure of a product to achieve the expected level of control when used according to the label recommendation for that pest species”^6^. Molecular mechanisms involved in insecticide resistance have been majorly attributed to behavioural and physiological resistance^7^. Behavioural resistance involves stimulus-dependent avoidance of insecticide doses that would prove lethal. Physiological resistance, on the other hand, involves target site point mutations, reduced insecticide penetration and enhanced enzymatic insecticide detoxification^7^.

Avermectins, diamides, organophosphates and pyrethroids are commonly used insecticide classes in the control of *P.* *absoluta* and therefore considered as the main focus in this study. Avermectins elicit their insecticidal action based on high affinity binding to glutamate-gated chloride channels and γ-aminobutyric acid (GABA_A_) receptors in the muscle and neural cells of the insects^8^. This causes irreversible paralysis and death of insects due to chloride ion influx within the nerve cells, which results in nerve impulse disruption^8^. Diamides target ryanodine receptors (RyRs) which play a key role in muscle contraction via the regulation of Ca^2+^ release within the sarcoplasmic reticulum of muscle cells^9^. Diamides modulate RyR upon binding, leaving calcium channels open inducing repeated muscle contraction and insect paralysis^9^. Insecticide resistance in *P.* *absoluta* has been reported for various chemical classes including organophosphates^10^, pyrethroids^11^, spinosyns^12^, cartap^13^, benzoylureas^14^, indoxacarb, avermectins^15^ and diamides^16^.

Though resistance to avermectins in *P.* *absoluta* has been reported, the underlying mechanism remains unknown. Meanwhile, reliance on diamides by farmers in Kenya as the most popular chemical control is still high, posing a greater risk for insecticide resistance selection. Another class of insecticides commonly used are organophosphates, which are neurotoxic synthetic insecticides that target the acetylcholinesterase (AChE) enzyme’s active site, causing irreversible inactivation. Consequently, the neurotransmitter acetylcholine builds up at the nerve junction resulting in uncontrolled nervous stimulation and death^17^. Insensitivity of AChE to organophosphates due to one or more point mutations of the *ace1* gene, which encodes AChE, has been reported in various insect species such as *Aedes* *aegypti,* *Drosophila* *melanogaster* and *Anopheles* *gambiae*^18^. Reduced sensitivity to organophosphates in *P.* *absoluta* has been attributed to a single point mutation (A201S) in the *ace-1* gene^10^. The frequently used pyrethroids target voltage-gated sodium channels by modulating the gating kinetics slowing down the closing of the channels and causing continuous conduction of sodium ions, which results in continuous nerve stimulation and death^19^. A common known resistance mechanism to pyrethroids in arthropods is via knock-down resistance (*kdr*) point mutation in the *para-*type voltage gated-sodium channel protein resulting in reduced sensitivity to pyrethroids^17^. The most common *kdr* mutation involves a single point mutation of leucine to phenylalanine (L1014F) substitution in domain IIS6 of the insect’s voltage-gated-sodium channel.

Furthermore, secondary enhanced mutations known as *super-kdr* have been reported in the domain IIS4-S5 linker, including M918T and T9291 in arthropods^17^. Therefore, exploring the knowledge of molecular mechanisms driving diamide and avermectin resistance selection in the Kenyan *P.* *absoluta* population is paramount and imperative to facilitate effective pest control and as a necessary tool in insecticide resistance management. To achieve this, this study sought to elucidate the gene expression profile of diamides and avermectins in parental and eighth-generation *P.* *absoluta-*exposed populations through RNA sequencing (RNA-Seq). Furthermore, we analysed the differential expression profiles of *ace1* and *para*-type voltage-gated sodium channel gene (VGSG) within first to eighth generations of diamide and avermectin-selected *P.* *absoluta* populations to identify cross-resistance of pyrethroids, diamides and avermectins in *P.* *absoluta* adults from Kenya.

## Results

### Insecticide bioassays

**Figure 1:**
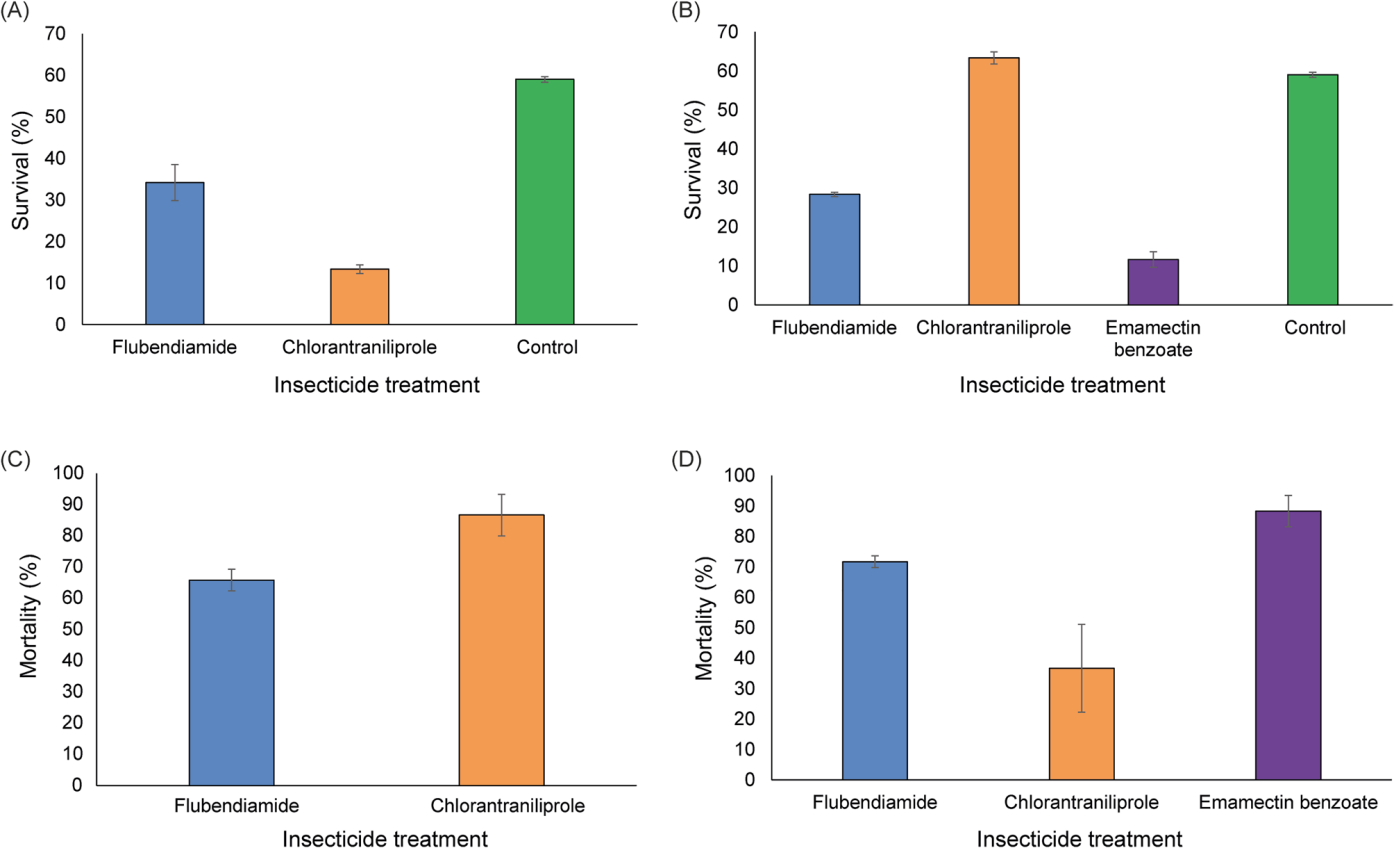
Responses of *Phthorimaea* *absoluta* individuals exposed to different spraying regimes after nine (9) days of exposure. (**A**) Survival of *Phthorimaea* *absoluta* individuals under direct insecticide sprays, (**B**) Survival of *Phthorimaea* *absoluta* individuals under indirect insecticide sprays, (**C**) Mortality of *Phthorimaea* *absoluta* individuals under direct insecticide sprays and (**D**) Mortality of *Phthorimaea* *absoluta* individuals under indirect insecticide sprays.

*Phthorimaea* *absoluta* adults were selected for this bioassay to emulate conditions in open fields and greenhouse insecticide spraying practices, which may lead to resistance development. *Phthorimaea* *absoluta* adult survivors from the parental generation were obtained in all bioassays except in the direct spraying scenario of emamectin benzoate insecticide, where 100% mortality was recorded nine days post-exposure. In the case of direct spraying of flubendiamide, a total of 34% of the moths survived after nine days, while 13% of the moths survived from direct spraying of chlorantraniliprole (Fig. 1A). In the indirect spraying of flubendiamide scenario, 28% of adult insects survived after nine days, while 63% survived the indirect spraying of chlorantraniliprole (Fig. 1B). The least amount of adult moth survival (12%) was observed after the indirect spraying of emamectin benzoate (Fig. 1B). The direct spraying of Chlorantraniliprole resulted in 86.7% mortality, while direct spraying of flubendiamide resulted in 65.8% percentage mortality (Fig. 1B). Furthermore, insect mortality showed the highest percentage in indirect emamectin benzoate (88%) compared to the other treatments (Fig. 1D). Flubendiamide indirect spraying resulted in 71.7% mortality, while chlorantraniliprole indirect spraying showed the least percentage mortality of 36.7% (Fig. 1D). Furthermore, across the generations (F1 to F8), survival ranged between 30 to 98% for the direct spraying scenario (Supplementary Fig. S1a), and for the indirect spraying scenario, survival ranged between 25 to 96% (Supplementary Fig. S1b).

### Differential gene expression analysis of *Phthorimaea absoluta*

**Figure 2:**
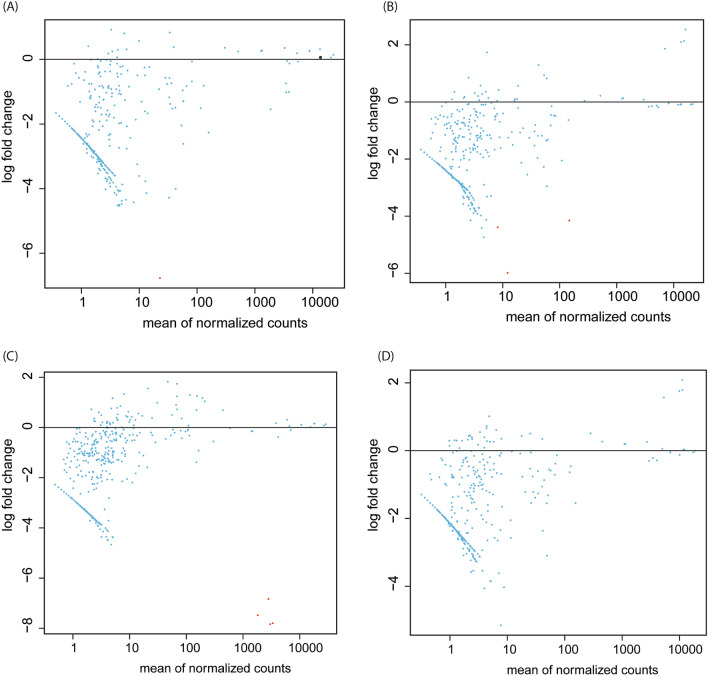
MA plots displaying the log fold-change compared with mean expression of differentially expressed genes in parental and eighth generations of Phthorimaea absoluta directly sprayed with (**A**) Chlorantraniliprole (parental generation), (**B**) Chlorantraniliprole (8th generation), (**C**) Flubendiamide (parental generation) and (**D**) Flubendiamide (8th generation) generated in DESeq2, with default log fold-change thresholds of − 1 and 1.

**Figure 3:**
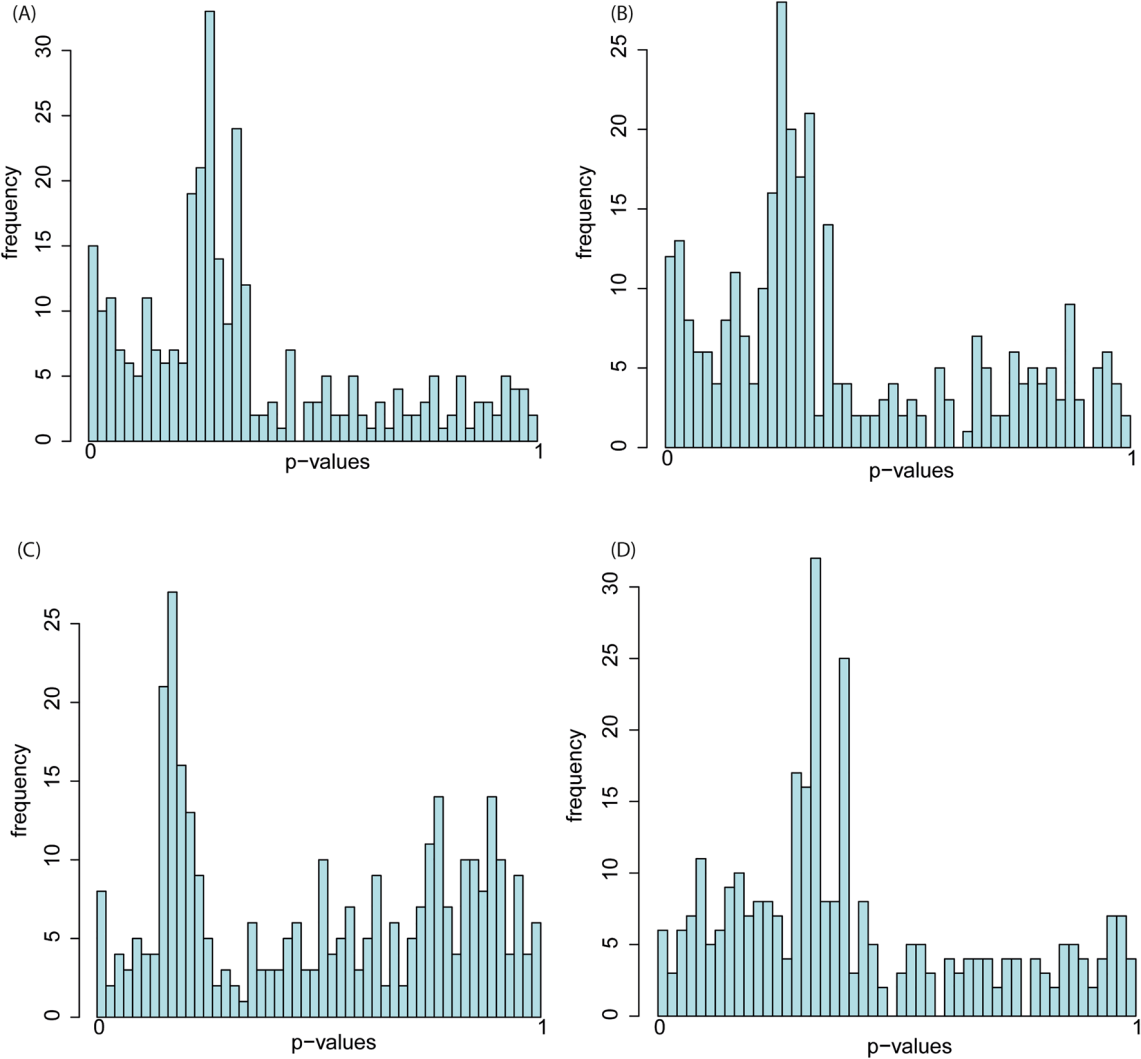
P-value distribution of differentially expressed genes in parental and eighth generations of Phthorimaea absoluta directly sprayed with (**A**) Chlorantraniliprole (parental generation), (**B**) Chlorantraniliprole (8th generation), (**C**) Flubendiamide (parental generation) and (D) Flubendiamide (8th generation) generated in DESeq2.

The transcriptomic analyses showed no significantly differentially expressed genes obtained from the three insecticides in parental and F8 samples under the indirect spraying scenario. On the other hand, the direct spraying scenario of flubendiamide and chlorantraniliprole (parental and F8 samples) had 82 significant DE genes. Out of the 82 significant DE genes, 77 genes (94%) were down-regulated, while five genes (6%) were up-regulated (Fig. 2). The chlorantraniliprole parental generation population had 31 significant differentially expressed genes, all down-regulated with log-fold change values ranging from negative two to negative six (Fig. 2A; Supplementary Table S1a). The chlorantraniliprole F8 population had 30 significantly differentially expressed genes, of which four were up-regulated and 26 were down-regulated (Fig. 2B; Supplementary Table S1b). However, the flubendiamide parental generation population had a total of 11 significant differentially expressed genes, with all of which were down-regulated, ranging from negative three to negative seven log-fold change values (Fig. 2C; Supplementary Table S1c). Additionally, the flubendiamide F8 population revealed 10 significantly differentially expressed genes, of which nine were down-regulated and one was up-regulated (Fig. 2D; Supplementary Table S1d). Directly sprayed chlorantraniliprole *P.* *absoluta* populations (both parental and F8) had a higher number of DEs than flubendiamide directly sprayed populations. A majority of expressed genes, as seen from the p-value distribution, ranged below 0.5 in both chlorantraniliprole and diamide-exposed parental and F8 *P.* *absoluta* populations (Fig. 3).

### Functional annotation of differentially expressed transcripts

**Figure 4:**
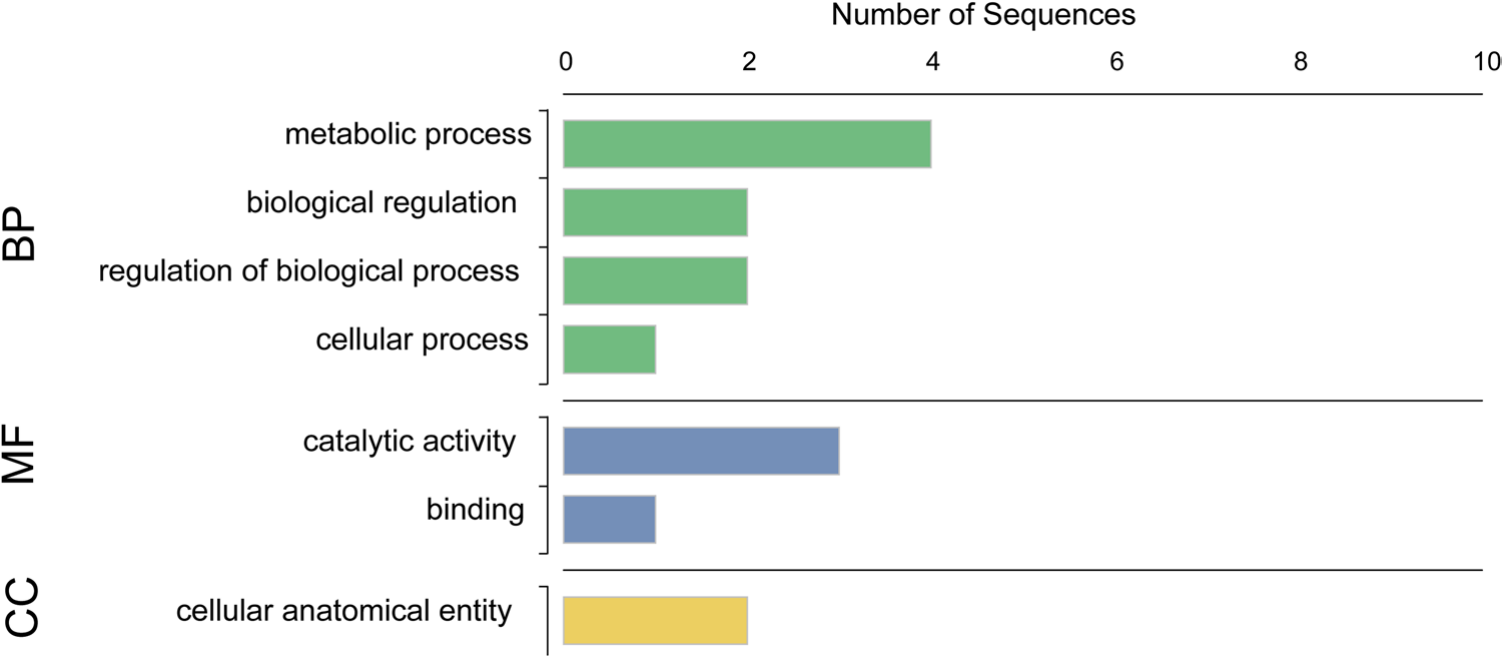
Distribution of functionally annotated gene sequences in differentially expressed genes in diamide-resistant *Phthorimaea absoluta* populations. *BP* Biological processes; *MF* Molecular function; *CC* Cellular component.

The Blastx results revealed eight hypothetical proteins, seven uncharacterized proteins and six proteins with specified descriptions (Supplementary Table S2). A total of seven sequences were successfully mapped, and the gene ontology annotation revealed three enzymatic activities, two integral components of the membrane and two involved in the regulation of the nitrogen compound metabolic process (Supplementary Table S3). Based on molecular functions, two transcripts displayed hydrolase catalytic function, while one transcript was involved in transferase catalytic function. Cellular component annotation revealed two transcripts involved in the integral component of membrane function. Biological process annotation revealed two transcripts involved in the regulation of macromolecule metabolic process (Fig. 4).

### RNA-Seq validation of differentially expressed genes using RT-qPCR

**Figure 5:**
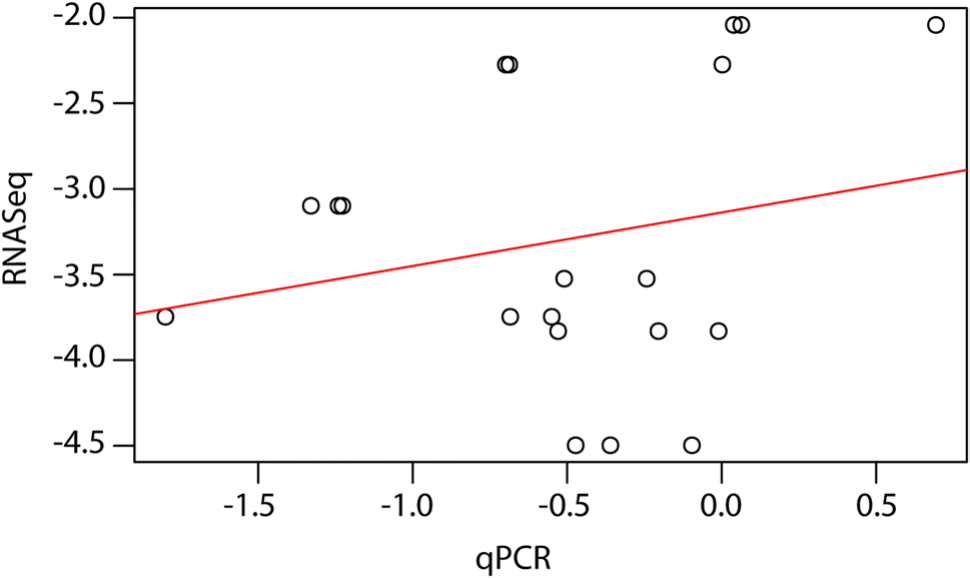
Pearson correlation plot of differentially expressed genes obtained from RNA-Seq and RT-qPCR for transcriptome validation.

The comparison of fold change expression levels of the seven down-regulated transcript candidates obtained in RNA-Seq and assessed using RT-qPCR, resulted in a Pearson correlation coefficient of 0.21, depicting a positive correlation and valid transcriptome (Fig. 5).

### A*ce1* and *para*-type voltage-gated sodium channel gene expression

**Figure 6:**
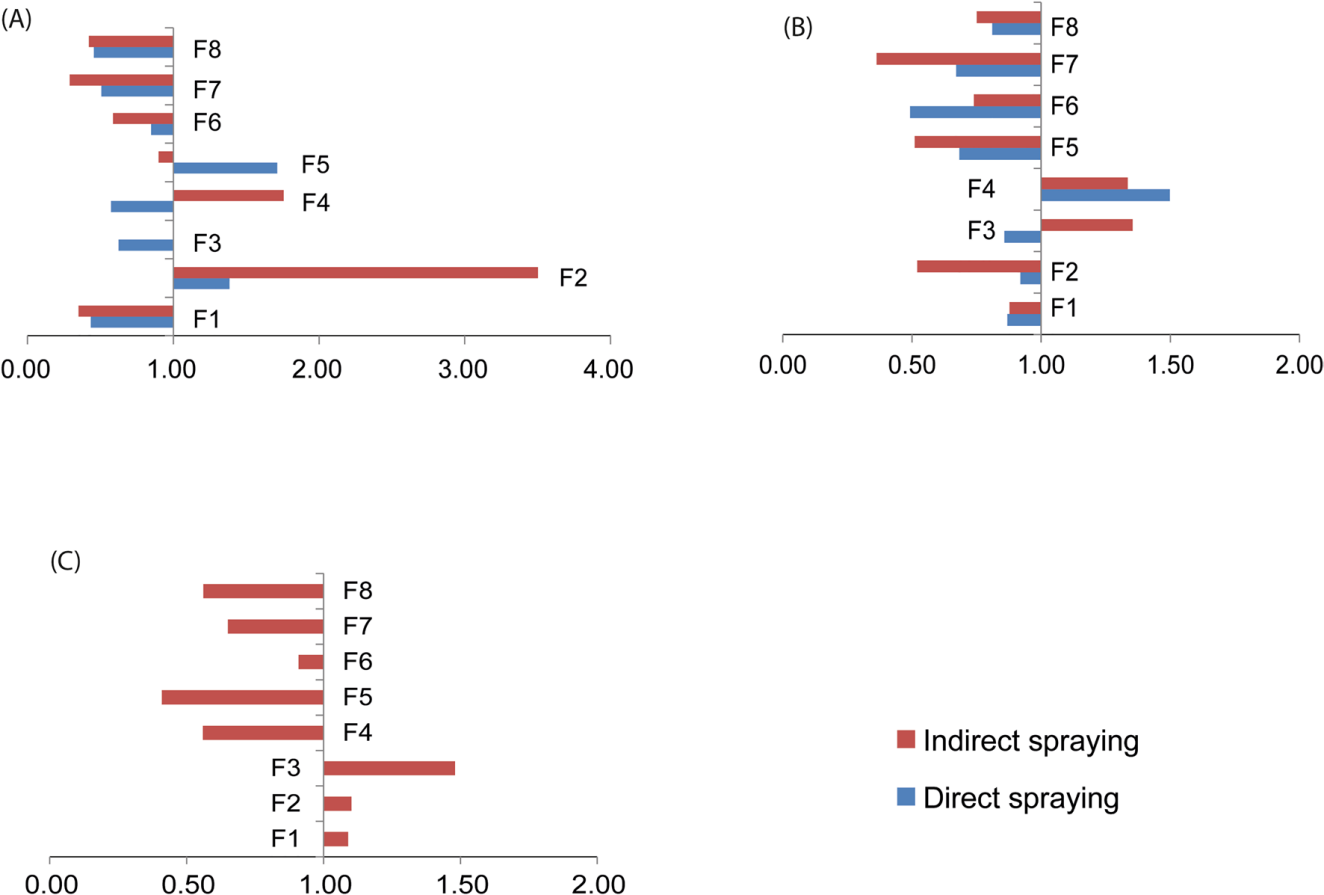
Expression of Ace1 gene in generations of *Phthorimaea absoluta* populations subjected to (**A**) Chlorantraniliprole, (**B**) Flubendiamide, and (**C**) Emamectin benzoate insecticides under direct and indirect spraying regimes. Values >1 show up-regulation of the gene and values <1 show down-regulation of the gene.

The qRT-PCR assays revealed the presence of *ace1,* and *P.* *absoluta* *para*-type voltage-gated sodium channel genes in all F1 to F8 progeny of diamide (chlorantraniliprole and flubendiamide) and avermectin (emamectin benzoate) selected *P.* *absoluta* populations*.* The directly sprayed chlorantraniliprole screened populations revealed an up-regulation of the *ace1* gene in the F4 generation only, while the remainder of the generations showed down-regulation of the same gene (Fig. 6A). The indirectly sprayed chlorantraniliprole screened populations showed an up-regulation of *ace1* gene in F3 and F4 generations while the rest of the generations showed down-regulation of the same gene (Fig. 6A). Across the directly sprayed flubendiamide selected population for *ace1* gene, up-regulation of *ace1* gene was observed in the F2 and F5 generations while down-regulation of the *ace1* gene was observed in the remainder F1, F3, F4, F6, F7 and F8 generations. Across the indirectly sprayed flubendiamide selected population of the *ace1* gene, up-regulation was observed in F2 and F4 generations, while down-regulation was observed in the remaining F1, F3, F5, F6, F7 and F8 generations (Fig. 6B). The indirectly sprayed emamectin benzoate screened population revealed up-regulation of *ace1* gene in F1, F2 and F3 generations, while the remainder of the generations showed down-regulation of *ace1* (Fig. 6C)*.*

**Figure 7:**
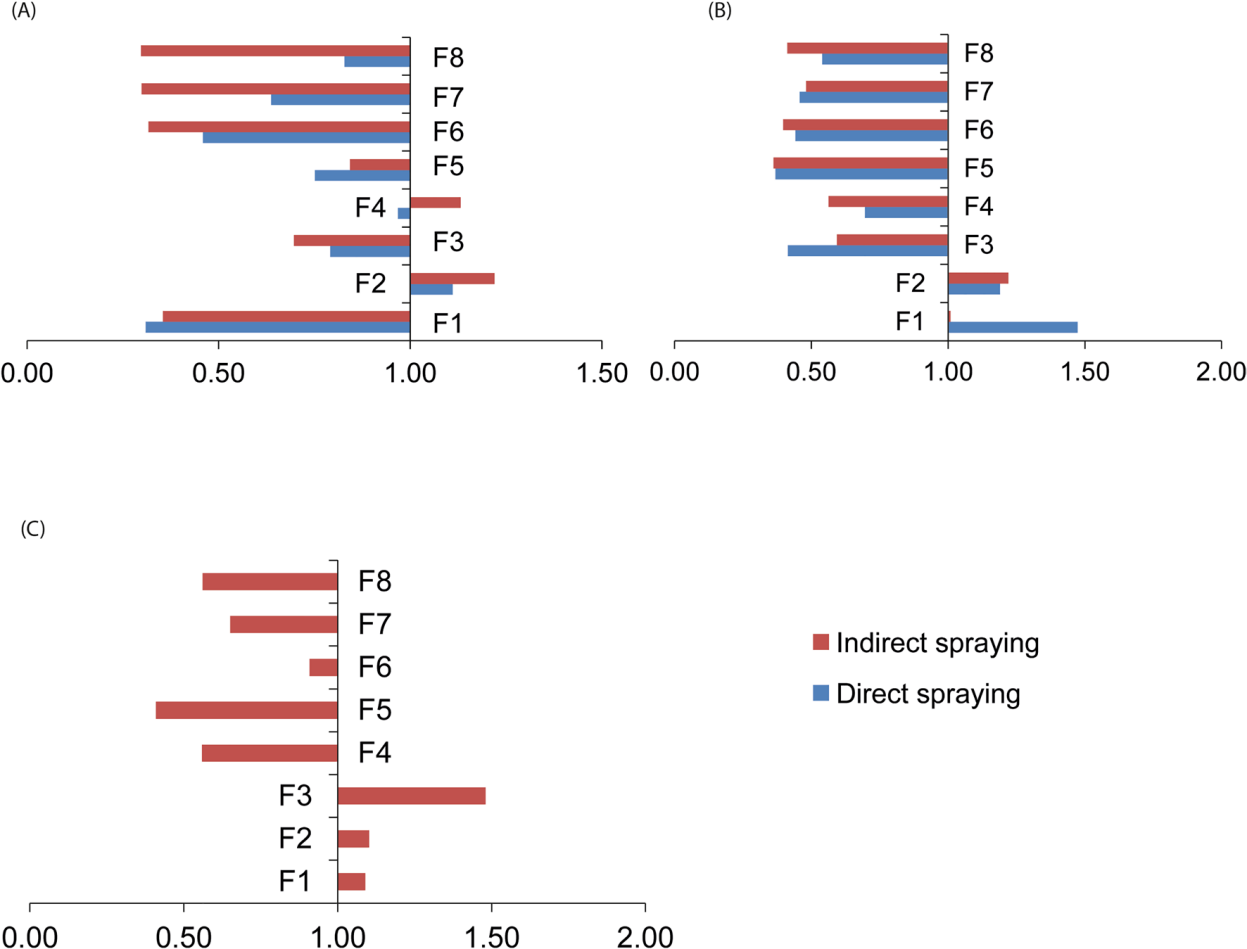
Expression of para-VGSG gene in generations of *Phthorimaea absoluta* populations subjected to (**A**) Chlorantraniliprole, (**B**) Flubendiamide, and (**C**) Emamectin benzoate insecticides under direct and indirect spraying regimes. Values >1 show up-regulation of the gene and values <1 show down-regulation of the gene.

The directly sprayed chlorantraniliprole screened population revealed up-regulation of *para* VGSG gene in F1 and F2 generations while down-regulation occurred in the remaining generations. Similarly, indirectly sprayed chlorantraniliprole screened populations revealed up-regulation of the *para* VGSG gene in F1 and F2 generations while down-regulation occurred in the remaining generations (Fig. 7A). In addition, the directly sprayed flubendiamide screened population depicted up-regulation of the *para* VGSG gene in F2 generation while down-regulation was observed in the remainder generations. The indirectly sprayed flubendiamide screened population revealed up-regulation of *para* VGSG in F2 and F4 generations, while down-regulation was observed in the remaining generations (Fig. 7B). Indirectly sprayed emamectin benzoate screened populations revealed up-regulation of *para* VGSG in F2 generation while down-regulation was observed in the rest of the generations (Fig. 7C).

## Discussion

In this study, we sought to gain insights into the mechanisms driving diamide and avermectin resistance selection in the Kenyan *P.* *absoluta* populations*,* as these two classes of pesticides are recommended by the Pest Control Products Board (PCPB) Kenya for *P.* *absoluta* management. We further screened the diamide and avermectin-resistant populations identified for resistance to organophosphates and pyrethroids, which target the *ace1* and *para*-type voltage-gated sodium channel gene (VGSG) in eight generations (F1–F8) of adult *P.* *absoluta* populations. The analysis conducted was two-fold. First, it was aimed at exploring genes targeted by diamides and avermectins through RNA sequencing and transcriptomic analyses comparing the parental and F8 adult *P.* *absoluta* generations only. Secondly, we explored the expression levels of *ace1* and para-type voltage-gated sodium channel gene (VGSG) genes across eight generations (F1–F8) of diamide and avermectin-selected *P.* *absoluta* populations through RT-qPCR. This was to further investigate possible multiple resistance or cross-resistance to organophosphates (*ace1* targets) and pyrethroids (VGSG targets) in *P.* *absoluta* populations.

Our study's choice of adult stages was made to emulate conditions in open fields and greenhouse insecticide spraying practices, which may lead to resistance development in the adult stages of the pest. Furthermore, larval and adult stages have been reported to show relatively similar responses to most insecticides tested in *Spodoptera* *littoralis*^20^. Based on the survivors obtained from the three insecticides (emamectin benzoate, flubendiamide and chlorantraniliprole) bioassays, our results showed that insecticide resistance selection was present within the obtained *P.* *absoluta* populations. This could be attributed to the heavy reliance on insecticide use and misuse by tomato farmers within open fields and greenhouses, resulting in gradual insecticide resistance selection^4^. The indirect spraying scenario presented survivors for all three insecticides sprayed as compared to the direct spraying scenario, which had no survivors for emamectin benzoate. Emamectin benzoate mode of insecticide resistance in *P.* *absoluta* could, therefore, be attributed to behavioural resistance, which is stimulus-dependent^7^. Spraying tomato plants with insecticides and later introducing them to the moths (indirect application) elicited hypersensitivity to the insecticide hence as nocturnal moths, they fled from the stimulus resulting in resistance selection. Insecticide resistance genes in insects are thought to be pre-adaptive, meaning they existed already in low frequencies in earlier populations as heterozygous phenotypes either prior to insecticide use or upon selection by other toxicants^21^. Furthermore, the survival rates of the adults across the generations provided evidence of the resistance to organophosphates and pyrethroids within the populations, as the percentage of treated insects which survived across the generations was comparable. The Rearing of *P.* *absoluta* to the 8th generation facilitated inbreeding which resulted in homozygosity of genes related to insecticide resistance hence establishing the expression of recessive alleles. Inbreeding effects are shown to be negligible after the 8th generation^22^. In relation to significantly differentially expressed (DE) genes across parental and F8 generations (in both directly and indirectly sprayed scenarios), flubendiamide expressed a lower percentage of DE genes (25.6%) compared to chlorantraniliprole (74.4%). This could be attributed to the pattern of diamide use; suggesting overuse of chlorantraniliprole within the greenhouse and open-field tomato farms hence increasing insecticide resistance selection pressure^4^. To further investigate the expression levels of *ace1* and *kdr/skdr* genes across F1–F8 generations of diamide and avermectin-selected *P.* *absoluta* population, we conducted Quantitative Reverse Transcription PCR on survivors obtained from the first to the eighth generations. The regions amplified within the ace*1* and *para*-type voltage-gated sodium channel gene contained known mutation sites; A201S and *kdr/skdr*, respectively. Based on the expression profiles obtained, the genes screened were not only present but were stable across F1–F8 generations in both directly and indirectly sprayed scenarios of diamide and avermectin-selected *P.* *absoluta* populations. Point mutations in the *ace-1* gene have been linked to AChE insensitivity and organophosphate resistance in insects^18^. In *P.* *absoluta*, sequencing of the *ace-1* gene revealed a single point mutation (A201S) of alanine to serine substitution believed to confer organophosphate resistance^10^. Point mutations in the domain II of the *para-*type voltage-gated sodium channels known as knock down resistance (*kdr*) or *skdr* (super kdr) have been attributed to confer pyrethroid insecticide resistance^17^. Sequencing of the *para-*type sodium channel IIS4–IIS6 region in *P.* *absoluta* revealed *kdr*-type mutation L1014F and two *skdr-*type mutations; M918T and T9291 that confer pyrethroid resistance^11^.

The functional annotation of the DE genes obtained in our study did not reveal the presence of ryanodine receptor proteins. Therefore, target-site mutations as a potential resistance mechanism for diamides were ruled out in this study, as mutations in G4903E of the ryanodine receptors have previously been attributed to diamide resistance in *P.* *absoluta*^16^. On the other hand, one transferase and two hydrolase transcripts were identified as detoxification enzymes in relation to insecticide resistance mechanisms, with significant down-regulation observed in this study. The role of detoxification enzymes in resistance development has been reported in *P.* *absoluta* populations^23^. The involvement of enhanced activity of cytochrome P450-dependent monooxygenases and esterases was reported in the resistance of *P.* *absoluta* populations to organophosphates (diazinon)^24^. Similarly, enhanced cytochrome P450-dependent monooxygenase and GST activities were observed in *P.* *absoluta* resistance to pyrethroids^25^. A significant difference in mean activity levels of glutathione S-transferase was reported between chlorantraniliprole-susceptible and resistant populations of *P.* *absoluta*^23^.

Our results further showed that *P.* *absoluta* genes encoding glutathione S-transferase and two hydrolases obtained from the transcriptomic profiling were significantly down-regulated. Hydrolases, glutathione S-transferase (GSTs) and cytochrome P450 monooxygenases have been identified in insects as the three key enzyme families in metabolic resistance mechanism^7^. Metabolic resistance employs the insect’s metabolic enzymes to detoxify insecticides to non-toxic or less toxic substances by increasing the levels of detoxification enzymes^26^. Esterases (Enzyme Commission number: EC 3.1) are phase I detoxification hydrolases that break ester bonds by adding water to yield alcohol and acid^7^. Two types of esterases namely; carboxylesterases and phosphatases, are majorly involved in insecticide detoxification^7^. Glutathione S-transferase (Enzyme Commission number: EC 2.5.1.18) facilitates the conjugation of electrophilic substrates with the thiol group of reduced glutathione (GSH) hence releasing more water soluble products for excretion and neutralizing highly reactive nucleophiles^27^. Up-regulation of genes encoding detoxification enzymes such as GSTs and carboxylesterases is linked to insecticide resistance selection in insects as observed in chemical exposed *P.* *absoluta* populations^26^. Nevertheless, down-regulation of GST and hydrolase genes upon exposure to insecticides has been cited in insect species such as the green peach aphid, *Myzus* *persicae* (Sulzer) (Hemiptera: Aphididae) and the Colorado potato beetle, *Leptinotarsa* *decemlineata* Say (Coleoptera: Chrysomelidae)^28,29^. In *M.* *persicae* resistant populations, GST genes (111036096 & 111036826) were down-regulated upon exposure to imidacloprid^28^. Down-regulation of genes encoding GSTs has been mentioned in *L.* *decemlineata* specifically LdGSTe4 and LdGSTe6, upon exposure to endosulfan, fipronil and cyhalothrin insecticides^29^. This phenomenon was attributed to the fact that insecticide pressure caused an up-regulation of specific GST subsets resulting in increased GST enzymatic activity^29^. However, increased enzymatic activity also resulted in the exhaustion of glutathione which could potentially cause harm to the insects. Consequently, down-regulation of another GST subset functions as an additional response to insecticide pressure^29^. Based on the mentioned insecticide response, the down-regulation of *P.* *absoluta* genes encoding GST and two hydrolases obtained from the transcriptomic profiling in our study could be therefore attributed to the same mechanism. Bautista et al.^28^ reported reduced expression of GSTs and carboxylesterases in *Plutella* *xylostella* (Linnaeus) (Lepidoptera: Plutellidae) but revealed increased cytochrome P450 and chemosensory protein expression. The study hypothesized the observations as an insecticide pressure adaptation based on energy trade-off to facilitate energy allocation to the most efficient detoxification genes upon insecticide pressure^30^. Similarly, our study showed a similar trend involving reduced expression of annotated GST and two carboxylesterases. However, further investigation may be required to identify the preferred energy-proficient detoxification genes involved in *P.* *absoluta* insecticide resistance.

## Conclusion

In conclusion, we identified differentially expressed genes in the directly sprayed diamide insecticides in both parental and F8 generations of *P.* *absoluta*. Indirect spraying showed that insecticide resistance was conferred mainly through altered behavioural response but showed a lack of differentially expressed genes. Our gene expression profiling revealed an increased down-regulation of 75% and 82.5%, in the genes linked to organophosphate and pyrethroid resistance respectively, across the F1–F8 diamide and avermectin-screened *P.* *absoluta* populations. Additionally, we found that the some of the mechanisms involved in diamide insecticide resistance were linked to detoxification enzymes, namely; transferases and hydrolases in Kenyan *P.* *absoluta* populations. The transcriptome information obtained in this study offers genomic resources for deeper research in the diamide and avermectin resistance mechanisms of *P.* *absoluta,* including future work on the functions of metabolic detoxification in *P.* *absoluta* through RNA interference studies and mechanisms driving cross-resistance of *P.* *absoluta* to the four classes of insecticides (avermectins, diamides, organophosphates and pyrethroids)*.* As our study only screened for the presence of the genes for resistance to pyrethroids and organophosphates in established diamide and avermectin-resistant *P.* *absoluta*, mortality data was not obtained from the F1–F8 generations since they were not subjected to insecticide treatment. Rather, they were allowed to complete their natural lifecycle while we screened them for pyrethroid and organophosphate resistance. Therefore, future studies on the mechanisms driving resistance of *P.* *absoluta* to organophosphates and pyrethroids will provide deeper insights into the resistance of *P.* *absoluta* to these classes of insecticides. Furthermore, the knowledge generated could serve to guide policies on insecticide use and in the effective management and monitoring of *P.* *absoluta* insecticide resistance. This will hence promote the use of effective insecticide chemistries and alternative management strategies such as biopesticides and biorationals, which are sustainable and eco-friendly to manage the pest, consequently reducing insecticide misuse/over-use in *P.* *absoluta* control.

## Methods

### Insect samples

*Phthorimaea* *absoluta* adults were initially obtained from infested tomato fields in Mwea (0° 36′ 31.3″ S 037° 22′ 29.7″ E) region of Kenya (June 2019) and used to establish a steady laboratory colony at the Animal Rearing and Quarantine Unit (ARQU) of the International Centre of Insect Physiology and Ecology (*icipe*)*.* They were reared in sleeved Perspex cages (40 cm × 40 cm × 45 cm) sustained at 28 ± 1 °C and a photoperiod of L12: D12. The moths were fed on 10% honey solution droplets coated on the top interior of each cage^31^. To facilitate female oviposition, four potted (4-week-old) tomato plants (*Solanum* *lycopersicum* L. cv. “Money maker”) were introduced into the cages. After 48 h of exposure, the potted plants were removed and placed on a separate bench area for egg hatching and subsequent larval leaf mining. After 3 days, the larvae-infested leaves were pruned off the plants and placed into plastic containers lined with paper towels and sealed with netting-attached lids for ventilation. The larvae were fed on a daily supply of fresh tomato leaves until pupation. Upon adult emergence, they were released from the ventilated plastic containers into exposure cages. Insect rearing was maintained for five generations before the bioassays were conducted.

### Insecticide bioassays

Three recommended insecticides according to the Pest Control Products Board (PCPB-Kenya) were chosen for the insecticide resistance selection bioassays, namely; emamectin benzoate (benzoic acid;(1′R,2R,3S,4′S,6S,8′R,10′E,12′S,13′S,14′E,16′E,20′R,21′R,24′S)-2-[(2S)-butan-2-yl]-21′,24′-dihydroxy-12′-[(2R,4S,5S,6S)-4-methoxy-5-[(2S,4S,5S,6S)-4-methoxy-6-methyl-5-(methylamino)oxan-2-yl]oxy-6-methyloxan-2-yl]oxy-3,11′,13′,22′-tetramethylspiro[2,3-dihydropyran-6,6′-3,7,19-trioxatetracyclo[15.6.1.14,8.020,24]pentacosa-10,14,16,22-tetraene]-2′-one) (class: avermectin), flubendiamide (1-*N*-[4-(1,1,1,2,3,3,3-heptafluoropropan-2-yl)-2-methylphenyl]-3-iodo-2-*N*-(2-methyl-1-methylsulfonylpropan-2-yl)benzene-1,2-dicarboxamide) and chlorantraniliprole (5-bromo-*N*-[4-chloro-2-methyl-6-(methylcarbamoyl)phenyl]-2-(3-chloropyridin-2-yl)pyrazole-3-carboxamide) (class: diamide)^32^. For each insecticide, two spraying scenarios were employed, namely; direct spraying and indirect spraying, for the purposes of emulating open field and greenhouse insecticide spraying practices. In the case of direct spraying, a total of 30 newly emerged adult insects (15 males and 15 females) were aspirated into sleeved Perspex cages (40 cm × 30 cm × 30 cm) in four replicates. Three potted tomato plants (*Solanum* *lycopersicum* L. cv. “Moneymaker”) about 3 weeks old, were introduced into each of the cages. For each of the three insecticides, the formulations to be sprayed were prepared in a 1-l hand sprayer according to the manufacturer’s recommended specifications for *P.* *absoluta* control (0.2 ml flubendiamide in 1000 ml distilled water, 0.4 ml emamectin benzoate in 1000 ml distilled water and 0.3 ml chlorantraniliprole in 1000 ml distilled water). In each cage containing both tomato potted plants and moth adults, each insecticide was sprayed directly onto the plant and insects alike with approximately 250 ml of prepared insecticide suspension. For the indirect spraying scenario, tomato plants were first sprayed with approximately 250 ml of prepared insecticide suspension using a one-litre hand sprayer as described above. In each cage containing 30 adult moths, three insecticide-sprayed potted tomato plants were then introduced. A control experiment was similarly employed for each insecticide and spraying scenario using sterile distilled water. Each bioassay experiment was maintained at 28 ± 1 °C, photoperiod of L12: D12 and 50–60% relative humidity (RH). After 48 h exposure, the number of eggs laid in each insecticide treatment types, based on the direct and indirect spraying scenario, was recorded while each potted plant was carefully removed for subsequent egg hatching and larval leaf mining assessment. The total live and dead adult *P.* *absoluta* after 9 days was recorded for each insecticide for both direct and indirect spraying scenarios. Adult moths that survived after nine days (original generation) were stored in Eppendorf tubes at − 80 °C for further downstream analyses. Based on the eggs laid by the adult moths that survived the insecticide spraying, larvae-infested leaves were pruned off and placed into plastic containers lined with paper towels and sealed with netting-attached lids for ventilation. The larvae were fed on a daily supply of fresh tomato leaves until pupation. Upon adult emergence, they were released from the ventilated plastic containers into sleeved Perspex exposure cages (40 cm × 30 cm × 30 cm) as described above. The cycle was repeated until the eighth generation to establish the stability of insecticide resistance genes^22^. Survived adult moths from the original to eighth generation were stored in Eppendorf tubes at − 80 °C for further downstream analyses.

### Transcriptome profiling using nanopore RNA sequencing

Total RNA was extracted from stored original generation and F8 generation *P.* *absoluta* adult samples obtained from both direct and indirect emamectin benzoate, flubendiamide and chlorantraniliprole insecticide spraying using Isolate II RNA Mini Kit according to manufacturer’s instructions. RNA purity was ascertained using Nanodrop 2000/2000c Spectrophotometer. Random hexamer primers were used for cDNA synthesis using SuperScript^®^ IV First Strand cDNA Synthesis Reaction Kit (Invitrogen, Carlsbad, CA, USA) following the manufacturer’s instructions. Library preparation for direct sequencing of barcoded cDNA was performed using SQK-DCS109 with EXP-NBD104 and EXP-NBD114 (Oxford Nanopore Technologies). The cDNA samples were taken through end-prep repair followed by barcode ligation and adapter ligation according to the manufacturer’s specifications. The libraries were quantified using Nanodrop 2000/2000c Spectrophotometer. Sequencing was set for 21 h on an Oxford Nanopore Technologies MK1C sequencing device using the MinION flow cell (version R9.4.1), while live base calling was done during the sequencing.

### Identification of differentially expressed genes

Demultiplexing and base calling of fast5 files was performed using Guppy base caller 5.1.13 under the fast base calling model and the read filtering q-score was set at eight. For each dataset of parental and F8 generation (under direct and indirect spraying scenario for each insecticide), a pipeline was employed for analysis using Galaxy Australia web-based platform^33^. Passed concatenated Fastq reads were adapter trimmed by Porechop^34^, followed by the alignment of adapter-trimmed reads against the *P.* *absoluta* genome by Minimap2 aligner^35^. A total of six biological replicates per treatment and control samples were used to analyse the transcripts and identify the differentially expressed genes. Transcripts were assembled using the String Tie transcript assembler^36^. Gene count files obtained from the reference-guided transcript assembly were used for differential gene expression analysis using Deseq2^37^. We then classified transcripts as differentially expressed (DE) if they had a minimum of a two-fold change in expression and p-value < 0.05. Artemis genome viewer^38^ was used to manually extract identified DE genes in Fasta format, compiled the DE sequences and functional annotation using Blast2GO^39^ was carried out.

### Validation of RNA sequencing by reverse transcription quantitative real-time PCR

To ensure that our RNA-seq results can be independently reproduced, reverse transcription quantitative real-time PCR (RT-qPCR) was employed. A total of seven down-regulated gene candidates were selected from the RNA-Seq results. A total of 24 biological *P.* *absoluta* adult samples from similar experimental conditions as those from the transcriptome experiment were used for the RT-qPCR experiment. For each candidate gene, we used three biological replicates, including control samples. The RT-qPCR involved three technical replicates for each of the three biological replicates. Primers for each of the seven genes, together with the housekeeping genes used [glyceraldehydes 3-phosphate dehydrogenase (GAPDH) and 18S rRNA], were carefully designed using Primer-BLAST^40^ (Supplementary Table S4). Reactions were set in the QuantStudio™ 5 Real-Time PCR System Machine. Gene expression levels were normalized to the housekeeping genes (GAPDH and 18S rRNA), and relative gene expression was calculated based on the 2^–∆∆Ct^ method^41^. We compared fold change expression levels obtained in RNA-sequencing and qRT-PCR within the seven transcript candidates. Pearson Correlation analysis of the fold changes was conducted to ascertain the validity of transcriptome data.

### Gene expression profiling of *ace1* and *para*-type voltage-gated sodium channel genes

To screen for organophosphate and pyrethroid resistance by identifying the expression levels of *ace1* *a*nd *para*-type voltage-gated sodium channel gene (VGSG), genes across eight generations (F1–F8) of diamide and avermectin-selected *P.* *absoluta* populations, we employed Reverse Transcription real-time qPCR (RT-qPCR). One hundred and sixty-four (164) biological samples of *P.* *absoluta* adults obtained from the above insecticide bioassays were used for the RT-qPCR experiment. These comprised four biological replicates for each generation (i.e. F1–F8) for both the direct and indirectly sprayed populations (as described in the insecticide bioassay experiments), including the control samples. Mortality was not obtained as the F2–F8 generations (diamide and avermectin-resistant population) were not subjected to insecticide treatment. Rather, they were allowed to complete their natural lifecycle while we screened them for pyrethroid and organophosphate resistance to see if there was cross-resistance to the four classes of insecticides. However, data on the number of survivors that emerged in each generation was collected. Total RNA extractions were conducted using Isolate II RNA Mini Kit (Bioline, London, UK) according to the manufacturer’s instructions. RNA purity was determined using Nanodrop 2000/2000c Spectrophotometer (Thermo Fischer Scientific, USA). Subsequent cDNAs were prepared using High-Capacity cDNA reverse transcription kit (Applied Biosystems, Carlsbad, CA) as per the manufacturer’s specifications. The qPCR assay comprised of three technical replicates for the four biological replicates, including the controls was conducted. Primers were designed against the *P.* *absoluta* strain TA1 voltage-gated sodium channel (*para*) gene containing the segment IIS4–IIS6 domains known to harbour *kdr* mutation sites^42^. Similarly, primers were designed targeting the *ace1* gene region with previously cited mutation sites linked with organophosphate resistance^43^ (Table 1). The SensiFAST SYBR Hi-Rox kit (Bioline) master mix was used for the assay. Reactions were set in the QuantStudio™ 5 Real-Time PCR System Machine (ThermoFisher). Gene expression levels were normalized to the housekeeping genes (GAPDH and 18S rRNA)^44,45^, and relative gene expression was calculated based on the 2^–∆∆Ct^ method^41^.

**Table 1:** List of primers used in RT-qPCR expression profiling of *ace1* and *para*-type voltage-gated sodium channel genes.

Name	Sequence 5′–3′	Target	Source
Ace-F1	GAAGCGATCCAAAATCGAAG	*Phthorimaea* *absoluta* acetylcholinesterase (ace1) gene region	Zibaee et al., 2017
Ace-R1	TGCCTAATGTACCCCATTCA	*Phthorimaea* *absoluta* acetylcholinesterase (ace1) gene region	Zibaee et al., 2017
TA1-Fw	TGGTGGGAGTGTTGCATGTT	*Phthorimaea* *absoluta* strain TA1 voltage-gated sodium channel (para) gene	JQ701800.1 (NCBI GenBank)
TA1-Rv	GGTCTCCATCGGGAAAACGA	*Phthorimaea* *absoluta* strain TA1 voltage-gated sodium channel (para) gene	JQ701800.1 (NCBI GenBank)

### Supplementary Information

Supplementary Information.

## Data Availability

All other relevant data are within the paper and supplementary materials. Sequences generated from this study were deposited in the GenBank database (www.ncbi.nlm.nih.gov/genbank) under the BioProject: PRJNA987408.
