# Supplementary material for: Biochemistry and transcriptomic analyses of Phthorimaea absoluta (Lepidoptera: Gelechiidae) response to insecticides
Source: Sci Rep. 2024 Apr 4;14:7931. doi: 10.1038/s41598-024-58413-z (PMC10995152; doi:10.1038/s41598-024-58413-z)
Supplement: Supplementary file 1 — Supplementary Information. [file 41598_2024_58413_MOESM1_ESM.docx]

**Supplementary Material**

**Biochemistry and Transcriptomic Analyses of *Phthorimaea absoluta* (Lepidoptera: Gelechiidae) Resistance to Insecticides**

Samantha W. Karanu^1,2^, Inusa J. Ajene^1^, Elijah Lelmen^2^, Maureen A. Ong’onge^1^ Komivi S. Akutse^1,3^ and Fathiya M. Khamis^1*^

^1^International Centre of Insect Physiology and Ecology, Nairobi, Kenya

^2^Department of Biochemistry, Egerton University, Egerton, Kenya

^3^Unit for Environmental Sciences and Management, North-West University, Potchefstroom, 2520, South Africa

*Corresponding author email: [fkhamis@icipe.org](mailto:fkhamis@icipe.org)

**
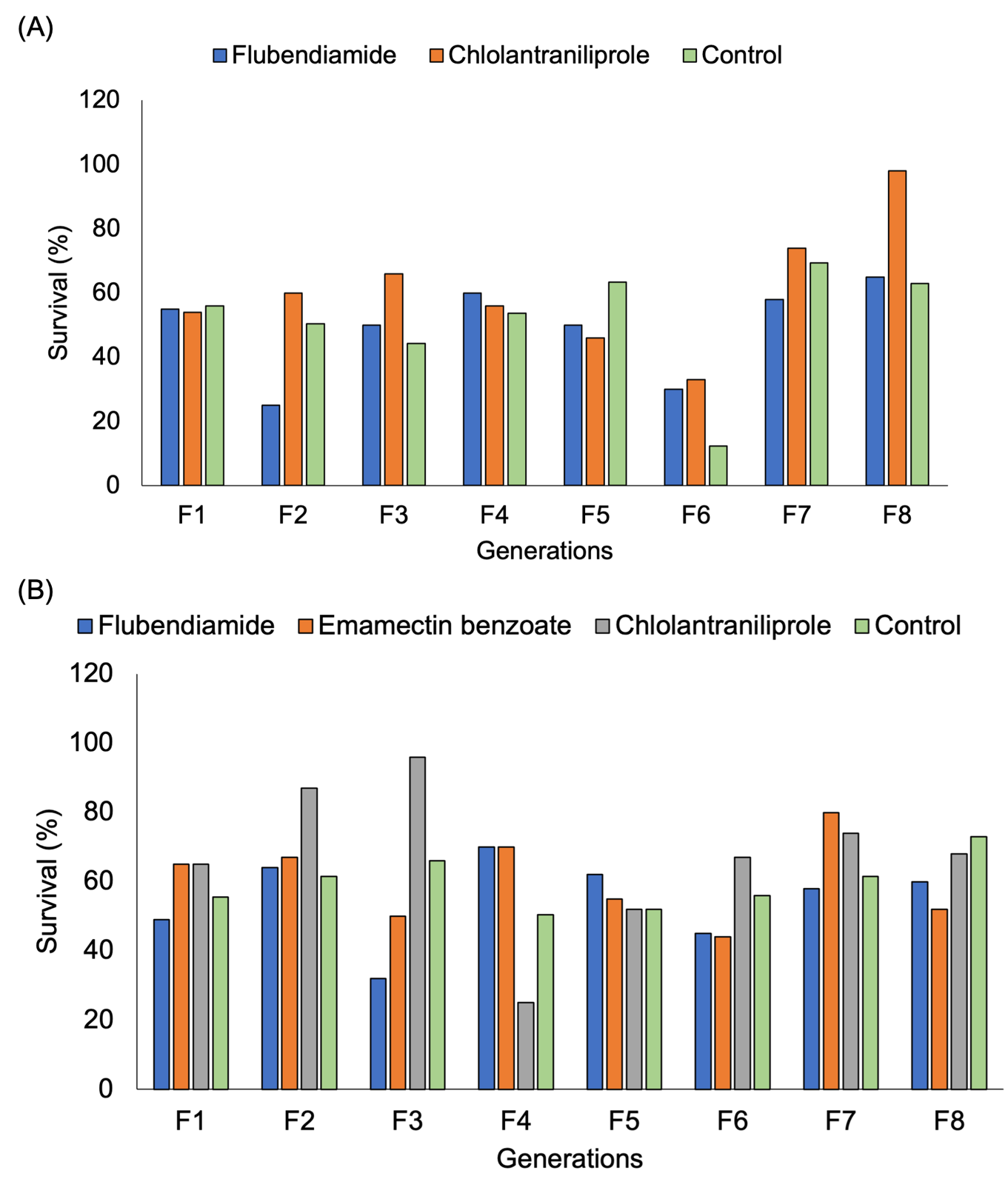
**

**Figure S1.** Survival of *Phthorimaea absoluta* adults exposed to flubendiamide, emamectin benzoate and chlorantraniliprole pesticides across eight generations under (a) direct insecticide sprays and (b) indirect insecticide sprays.

**Table S1. List of significantly differentially expressed genes from DESeq2**

**Table S1 (a). Chlorantraniliprole parental generation**

| **Gene ID** | **Base mean expression** | **log2(FC)** | **stdErr** | **wald_stats** | **P-value** | **P-adj** |
| --- | --- | --- | --- | --- | --- | --- |
| MSTRG.49 | 22.8158 | -6.7687 | 1.5086 | -4.4867 | 7.23E-06 | 0.0023 |
| MSTRG.234 | 8.0289 | -4.4230 | 1.4300 | -3.0930 | 0.0020 | 0.2703 |
| MSTRG.62 | 32.7559 | -4.2829 | 1.4217 | -3.0125 | 0.0026 | 0.2703 |
| MSTRG.10 | 4.5528 | -4.5293 | 1.6810 | -2.6945 | 0.0071 | 0.3263 |
| MSTRG.103 | 42.5956 | -4.0054 | 1.4434 | -2.7749 | 0.0055 | 0.3263 |
| MSTRG.149 | 4.2322 | -4.5237 | 1.6527 | -2.7372 | 0.0062 | 0.3263 |
| MSTRG.218 | 4.4468 | -4.4972 | 1.6845 | -2.6697 | 0.0076 | 0.3263 |
| MSTRG.506 | 13.3297 | -4.1377 | 1.5685 | -2.6380 | 0.0083 | 0.3263 |
| MSTRG.455 | 4.9684 | -4.4756 | 1.7388 | -2.5739 | 0.0101 | 0.3497 |
| MSTRG.132 | 4.9855 | -4.3774 | 1.7578 | -2.4902 | 0.0128 | 0.3632 |
| MSTRG.137 | 2.6171 | -4.0205 | 1.6137 | -2.4915 | 0.0127 | 0.3632 |
| MSTRG.152 | 4.0482 | -4.1888 | 1.7646 | -2.3738 | 0.0176 | 0.3936 |
| MSTRG.464 | 12.7630 | -3.7716 | 1.5586 | -2.4199 | 0.0155 | 0.3936 |
| MSTRG.85 | 10.0316 | -3.6073 | 1.5049 | -2.3971 | 0.0165 | 0.3936 |
| MSTRG.5 | 3.7397 | -4.1265 | 1.7580 | -2.3473 | 0.0189 | 0.3946 |
| MSTRG.247 | 4.6749 | -4.0666 | 1.8319 | -2.2198 | 0.0264 | 0.4422 |
| MSTRG.258 | 4.2708 | -4.0421 | 1.8259 | -2.2138 | 0.0268 | 0.4422 |
| MSTRG.419 | 3.1440 | -3.9325 | 1.7636 | -2.2298 | 0.0258 | 0.4422 |
| MSTRG.472 | 4.4145 | -4.0821 | 1.8238 | -2.2383 | 0.0252 | 0.4422 |
| MSTRG.24 | 9.2108 | -3.2455 | 1.4934 | -2.1733 | 0.0298 | 0.4489 |
| MSTRG.324 | 3.0879 | -3.8305 | 1.7796 | -2.1524 | 0.0314 | 0.4489 |
| MSTRG.477 | 3.9810 | -3.9432 | 1.8340 | -2.1500 | 0.0316 | 0.4489 |
| MSTRG.288 | 2.7587 | -3.7737 | 1.7729 | -2.1285 | 0.0333 | 0.4531 |
| MSTRG.160 | 3.3021 | -3.7467 | 1.8379 | -2.0385 | 0.0415 | 0.4810 |
| MSTRG.243 | 1.9327 | -3.5276 | 1.7243 | -2.0458 | 0.0408 | 0.4810 |
| MSTRG.365 | 2.7828 | -3.7095 | 1.7954 | -2.0661 | 0.0388 | 0.4810 |
| MSTRG.80 | 2.6045 | -3.6809 | 1.7855 | -2.0616 | 0.0393 | 0.4810 |
| MSTRG.317 | 53.7870 | -2.0425 | 1.0227 | -1.9971 | 0.0458 | 0.4886 |
| MSTRG.499 | 3.0203 | -3.6427 | 1.8428 | -1.9767 | 0.0481 | 0.4886 |
| MSTRG.76 | 3.1693 | -3.6935 | 1.8416 | -2.0055 | 0.0449 | 0.4886 |
| MSTRG.93 | 3.0015 | -3.6372 | 1.8426 | -1.9739 | 0.0484 | 0.4886 |

**Table S1 (b). Chlorantraniliprole F8 generation**

| **Gene ID** | **Base mean expression** | **log2(FC)** | **stdErr** | **wald_stats** | **P-value** | **P-adj** |
| --- | --- | --- | --- | --- | --- | --- |
| MSTRG.464 | 12.2103 | -5.9865 | 1.4775 | -4.0518 | 5.08E-05 | 0.0161 |
| MSTRG.27 | 147.1438 | -4.1547 | 1.1597 | -3.5825 | 0.0003 | 0.0538 |
| MSTRG.234 | 8.1665 | -4.3916 | 1.2662 | -3.4683 | 0.0005 | 0.0552 |
| MSTRG.10 | 4.6328 | -4.7395 | 1.5601 | -3.0379 | 0.0024 | 0.1689 |
| MSTRG.97 | 16061.3755 | 2.5351 | 0.8441 | 3.0031 | 0.0027 | 0.1689 |
| MSTRG.482 | 108.7318 | -2.0540 | 0.7003 | -2.9332 | 0.0034 | 0.1767 |
| MSTRG.137 | 2.7268 | -4.1470 | 1.5531 | -2.6702 | 0.0076 | 0.3009 |
| MSTRG.152 | 4.2768 | -4.4104 | 1.6527 | -2.6686 | 0.0076 | 0.3009 |
| MSTRG.352 | 59.3444 | -2.9537 | 1.1770 | -2.5096 | 0.0121 | 0.3820 |
| MSTRG.92 | 4.1606 | -3.9787 | 1.5761 | -2.5244 | 0.0116 | 0.3820 |
| MSTRG.102 | 2.4981 | -3.6307 | 1.7325 | -2.0956 | 0.0361 | 0.4698 |
| MSTRG.15 | 3.1782 | -3.7338 | 1.7633 | -2.1175 | 0.0342 | 0.4698 |
| MSTRG.160 | 3.3417 | -3.8293 | 1.7512 | -2.1866 | 0.0288 | 0.4698 |
| MSTRG.182 | 2.8859 | -3.6105 | 1.7723 | -2.0372 | 0.0416 | 0.4698 |
| MSTRG.243 | 2.0410 | -3.5706 | 1.6878 | -2.1156 | 0.0344 | 0.4698 |
| MSTRG.324 | 3.0786 | -3.8927 | 1.7023 | -2.2868 | 0.0222 | 0.4698 |
| MSTRG.351 | 52.4786 | -2.2742 | 1.0696 | -2.1261 | 0.0335 | 0.4698 |
| MSTRG.385 | 2.9001 | -3.6195 | 1.7712 | -2.0435 | 0.0410 | 0.4698 |
| MSTRG.455 | 5.3045 | -3.3161 | 1.5561 | -2.1311 | 0.0331 | 0.4698 |
| MSTRG.49 | 27.0732 | -2.5450 | 1.2265 | -2.0750 | 0.0380 | 0.4698 |
| MSTRG.499 | 3.0956 | -3.7143 | 1.7625 | -2.1075 | 0.0351 | 0.4698 |
| MSTRG.56 | 13403.9329 | 2.0952 | 0.8906 | 2.3525 | 0.0186 | 0.4698 |
| MSTRG.57 | 7024.9039 | 1.8611 | 0.9073 | 2.0511 | 0.0403 | 0.4698 |
| MSTRG.76 | 3.2048 | -3.7599 | 1.7589 | -2.1376 | 0.0326 | 0.4698 |
| MSTRG.80 | 2.6838 | -3.7431 | 1.7194 | -2.1770 | 0.0295 | 0.4698 |
| MSTRG.87 | 3.3959 | -3.8291 | 1.7546 | -2.1824 | 0.0291 | 0.4698 |
| MSTRG.93 | 3.2138 | -3.7693 | 1.7570 | -2.1453 | 0.0319 | 0.4698 |
| MSTRG.98 | 15226.8088 | 2.1350 | 0.8901 | 2.3985 | 0.0165 | 0.4698 |
| MSTRG.298 | 2.8061 | -3.5241 | 1.7834 | -1.9760 | 0.0482 | 0.5072 |
| MSTRG.82 | 2.7821 | -3.5411 | 1.7807 | -1.9886 | 0.0467 | 0.5072 |

**Table S1 (c). Flubendiamide parental generation**

| **Gene ID** | **Base mean expression** | **log2(FC)** | **stdErr** | **wald_stats** | **P-value** | **P-adj** |
| --- | --- | --- | --- | --- | --- | --- |
| MSTRG.98 | 3343.6422 | -7.7993 | 1.0827 | -7.2036 | 5.87E-13 | 1.98E-10 |
| MSTRG.56 | 3013.8100 | -7.8358 | 1.2769 | -6.1368 | 8.42E-10 | 9.46E-08 |
| MSTRG.97 | 2793.1433 | -6.8317 | 1.1039 | -6.1888 | 6.06E-10 | 9.46E-08 |
| MSTRG.57 | 1822.1217 | -7.4817 | 1.2619 | -5.9287 | 3.05E-09 | 2.57E-07 |
| MSTRG.102 | 3.1836 | -4.3579 | 1.8481 | -2.3580 | 0.0184 | 0.6760 |
| MSTRG.249 | 4.7524 | -4.6675 | 1.8860 | -2.4748 | 0.0133 | 0.6760 |
| MSTRG.345 | 2.7303 | -3.9129 | 1.9482 | -2.0085 | 0.0446 | 0.6760 |
| MSTRG.354 | 3.3974 | -4.1458 | 1.9293 | -2.1489 | 0.0316 | 0.6760 |
| MSTRG.468 | 3.0107 | -4.0571 | 1.9333 | -2.0985 | 0.0359 | 0.6760 |
| MSTRG.76 | 4.1325 | -4.4935 | 1.8961 | -2.3698 | 0.0178 | 0.6760 |
| MSTRG.93 | 4.0725 | -4.4787 | 1.8958 | -2.3625 | 0.0182 | 0.6760 |

**Table S1 (d). Flubendiamide F8 generation**

| **Gene ID** | **Base mean expression** | **log2(FC)** | **stdErr** | **wald_stats** | **P-value** | **P-adj** |
| --- | --- | --- | --- | --- | --- | --- |
| MSTRG.24 | 7.6896 | -5.1525 | 1.4475 | -3.5597 | 0.0004 | 0.1162 |
| MSTRG.10 | 3.9811 | -4.0618 | 1.5856 | -2.5617 | 0.0104 | 0.6167 |
| MSTRG.128 | 2.5391 | -3.5422 | 1.5969 | -2.2181 | 0.0265 | 0.6167 |
| MSTRG.137 | 2.3219 | -3.5812 | 1.5341 | -2.3344 | 0.0196 | 0.6167 |
| MSTRG.234 | 7.0980 | -3.6189 | 1.3679 | -2.6455 | 0.0082 | 0.6167 |
| MSTRG.258 | 3.6856 | -3.5438 | 1.6949 | -2.0908 | 0.0365 | 0.6167 |
| MSTRG.352 | 48.9586 | -3.0988 | 1.2727 | -2.4347 | 0.0149 | 0.6167 |
| MSTRG.46 | 5.0604 | -3.8534 | 1.6883 | -2.2825 | 0.0225 | 0.6167 |
| MSTRG.85 | 8.7419 | -4.0315 | 1.4717 | -2.7393 | 0.0062 | 0.6167 |
| MSTRG.97 | 11080.3394 | 2.0846 | 1.0421 | 2.0004 | 0.0455 | 0.6167 |

**Table S2. Transcriptomic BlastX results**

| **TAGS** | **SEQ NAME** | **DESCRIPTION** | **LENGTH** | **HITS** | **e-VALUE** | **SIM MEAN** |
| --- | --- | --- | --- | --- | --- | --- |
| INTERPRO, BLASTED | exon_MSTRG.102 | hypothetical protein SFRUCORN_021359 | 807 | 20 | 7.40E-32 | 81.04 |
| INTERPRO, BLASTED | exon_MSTRG.249 | hypothetical protein SFRURICE_013418 | 1842 | 1 | 2.33E-11 | 96.67 |
| INTERPRO, BLASTED | exon_MSTRG.93 | reverse transcriptase domain-containing protein | 892 | 20 | 7.20E-46 | 83.24 |
| INTERPRO, BLASTED | exon_MSTRG.49 | hypothetical protein SFRURICE_016287, partial | 919 | 20 | 8.11E-26 | 89.19 |
| INTERPRO, BLASTED | exon_MSTRG.234 | hypothetical protein SFRURICE_015825 | 1647 | 1 | 1.09E-31 | 58.52 |
| INTERPRO, BLASTED | exon_MSTRG.62 | uncharacterized protein LOC118272849 | 2048 | 20 | 0 | 77.87 |
| INTERPRO, BLASTED | exon_MSTRG.103 | hypothetical protein SFRUCORN_019536 | 3355 | 20 | 1.52E-21 | 80.34 |
| INTERPRO, BLASTED | exon_MSTRG.218 | unnamed protein product | 1393 | 20 | 3.88E-141 | 57.35 |
| INTERPRO, BLASTED | exon_MSTRG.464 | DDE superfamily endonuclease domain-containing protein | 1765 | 13 | 9.44E-24 | 93.1 |
| INTERPRO, BLASTED | exon_MSTRG.85 | uncharacterized protein LOC118268535 | 1410 | 2 | 5.37E-27 | 92.15 |
| INTERPRO, BLASTED | exon_MSTRG.419 | uncharacterized protein LOC118265113 | 915 | 20 | 1.06E-73 | 84.1 |
| INTERPRO, BLASTED | exon_MSTRG.24 | uncharacterized protein LOC118265113 | 1662 | 20 | 5.66E-80 | 83.5 |
| INTERPRO, BLASTED | exon_MSTRG.324 | hypothetical protein B5X24_HaOG201741 | 741 | 20 | 1.36E-140 | 83.99 |
| INTERPRO, BLASTED | exon_MSTRG.160 | uncharacterized protein LOC115440776 | 1218 | 20 | 5.99E-140 | 78.85 |
| INTERPRO, BLASTED | exon_MSTRG.317 | uncharacterized protein LOC118265175 | 1642 | 20 | 0 | 83.74 |
| INTERPRO, BLASTED | exon_MSTRG.352 | modifier of mdg4-like | 2500 | 20 | 1.24E-50 | 95.47 |
| INTERPRO, BLASTED | exon_MSTRG.482 | hypothetical protein SFRURICE_016287, partial | 4838 | 20 | 6.66E-24 | 89.19 |
| INTERPRO, BLASTED | exon_MSTRG.15 | hypothetical protein SFRURICE_016287, partial | 914 | 20 | 7.91E-26 | 89.25 |
| INTERPRO, BLASTED | exon_MSTRG.182 | hypothetical protein SFRUCORN_003575 | 1150 | 3 | 6.68E-19 | 100 |
| INTERPRO, BLASTED | exon_MSTRG.351 | protein tramtrack, beta isoform-like | 3718 | 20 | 3.17E-29 | 84.6 |
| BLASTED | exon_MSTRG.87 | Rho-related GTP-binding protein RhoC | 1411 | 2 | 3.98E-14 | 53.44 |
| INTERPRO, BLASTED | exon_MSTRG.298 | LINE-1 retrotransposable element ORF2 protein isoform X1 | 1055 | 20 | 2.18E-95 | 84.42 |
| INTERPRO, BLASTED | exon_MSTRG.82 | uncharacterized protein LOC118269018 | 1118 | 20 | 0 | 78.9 |

**Table S3. List of annotated transcripts**

| **SeqName** | **Description** | **#GO** | **GO IDs** | **GO names** | **enzyme codes** | **enzyme name** |
| --- | --- | --- | --- | --- | --- | --- |
| exon_MSTRG.218 | unnamed protein product | 3 | P:GO:0036211; F:GO:0016740; F:GO:0140096 | P:protein modification process; F:transferase activity; F:catalytic activity, acting on a protein | EC:2.7.1 | Transferring phosphorus-containing groups |
| exon_MSTRG.324 | hypothetical protein B5X24_HaOG201741 | 1 | C:GO:0016021 | C:integral component of membrane |  |  |
| exon_MSTRG.160 | uncharacterized protein LOC115440776 | 1 | C:GO:0016021 | C:integral component of membrane |  |  |
| exon_MSTRG.317 | uncharacterized protein LOC118265175 | 2 | P:GO:0016071; F:GO:0016787 | P:mRNA metabolic process; F:hydrolase activity | EC:3.1 | Acting on ester bonds |
| exon_MSTRG.352 | modifier of mdg4-like | 3 | P:GO:0051171; P:GO:0060255; P:GO:0080090 | P:regulation of nitrogen compound metabolic process; P:regulation of macromolecule metabolic process; P:regulation of primary metabolic process |  |  |
| exon_MSTRG.351 | protein tramtrack, beta isoform-like | 4 | P:GO:0051171; P:GO:0060255; P:GO:0080090; F:GO:0005515 | P:regulation of nitrogen compound metabolic process; P:regulation of macromolecule metabolic process; P:regulation of primary metabolic process; F:protein binding |  |  |
| exon_MSTRG.298 | LINE-1 retrotransposable element ORF2 protein isoform X1 | 1 | F:GO:0016787 | F:hydrolase activity | EC:3.1 | Acting on ester bonds |

**Table S3 (a). List of biological process gene ontologies (GO)**

| **GO ID** | **GO Name** | **GO Type** | **Parents (ACC)** | **Parents (Name)** | **Nodescore** | **#Seqs** | **Sequence Names** |
| --- | --- | --- | --- | --- | --- | --- | --- |
| GO:0008150 | biological_process | Biological Process |  |  | 1.47 | 4 | exon_MSTRG.351, exon_MSTRG.317, exon_MSTRG.218, exon_MSTRG.352 |
| GO:0050789 | regulation of biological process | Biological Process | GO:0065007, GO:0008150 | biological regulation, biological_process | 2.16 | 2 | exon_MSTRG.351, exon_MSTRG.352 |
| GO:0008152 | metabolic process | Biological Process | GO:0008150 | biological_process | 2.45 | 4 | exon_MSTRG.351, exon_MSTRG.317, exon_MSTRG.218, exon_MSTRG.352 |
| GO:0065007 | biological regulation | Biological Process | GO:0008150 | biological_process | 1.3 | 2 | exon_MSTRG.351, exon_MSTRG.352 |
| GO:0009987 | cellular process | Biological Process | GO:0008150 | biological_process | 0.05 | 1 | exon_MSTRG.317 |
| GO:0006807 | nitrogen compound metabolic process | Biological Process | GO:0008152 | metabolic process | 1.49 | 4 | exon_MSTRG.351, exon_MSTRG.317, exon_MSTRG.218, exon_MSTRG.352 |
| GO:0044237 | cellular metabolic process | Biological Process | GO:0009987, GO:0008152 | cellular process, metabolic process | 0.08 | 1 | exon_MSTRG.317 |
| GO:0044238 | primary metabolic process | Biological Process | GO:0008152 | metabolic process | 1.69 | 4 | exon_MSTRG.351, exon_MSTRG.317, exon_MSTRG.218, exon_MSTRG.352 |
| GO:0019222 | regulation of metabolic process | Biological Process | GO:0050789, GO:0008152 | regulation of biological process, metabolic process | 3.6 | 2 | exon_MSTRG.351, exon_MSTRG.352 |
| GO:0071704 | organic substance metabolic process | Biological Process | GO:0008152 | metabolic process | 1.07 | 4 | exon_MSTRG.351, exon_MSTRG.317, exon_MSTRG.218, exon_MSTRG.352 |
| GO:0034641 | cellular nitrogen compound metabolic process | Biological Process | GO:0006807, GO:0044237 | nitrogen compound metabolic process, cellular metabolic process | 0.13 | 1 | exon_MSTRG.317 |
| GO:0006139 | nucleobase-containing compound metabolic process | Biological Process | GO:0046483, GO:0034641, GO:0006725, GO:0044238, GO:1901360 | heterocycle metabolic process, cellular nitrogen compound metabolic process, cellular aromatic compound metabolic process, primary metabolic process, organic cyclic compound metabolic process | 0.22 | 1 | exon_MSTRG.317 |
| GO:0006725 | cellular aromatic compound metabolic process | Biological Process | GO:0044237 | cellular metabolic process | 0.13 | 1 | exon_MSTRG.317 |
| GO:1901564 | organonitrogen compound metabolic process | Biological Process | GO:0006807, GO:0071704 | nitrogen compound metabolic process, organic substance metabolic process | 0.36 | 1 | exon_MSTRG.218 |
| GO:1901360 | organic cyclic compound metabolic process | Biological Process | GO:0071704 | organic substance metabolic process | 0.13 | 1 | exon_MSTRG.317 |
| GO:0060255 | regulation of macromolecule metabolic process | Biological Process | GO:0019222, GO:0043170 | regulation of metabolic process, macromolecule metabolic process | 2 | 2 | exon_MSTRG.351, exon_MSTRG.352 |
| GO:0051171 | regulation of nitrogen compound metabolic process | Biological Process | GO:0019222, GO:0006807 | regulation of metabolic process, nitrogen compound metabolic process | 2 | 2 | exon_MSTRG.351, exon_MSTRG.352 |
| GO:0019538 | protein metabolic process | Biological Process | GO:0043170, GO:0044238, GO:1901564 | macromolecule metabolic process, primary metabolic process, organonitrogen compound metabolic process | 0.6 | 1 | exon_MSTRG.218 |
| GO:0046483 | heterocycle metabolic process | Biological Process | GO:0044237 | cellular metabolic process | 0.13 | 1 | exon_MSTRG.317 |
| GO:0080090 | regulation of primary metabolic process | Biological Process | GO:0019222, GO:0044238 | regulation of metabolic process, primary metabolic process | 2 | 2 | exon_MSTRG.351, exon_MSTRG.352 |
| GO:0043170 | macromolecule metabolic process | Biological Process | GO:0071704 | organic substance metabolic process | 1.78 | 4 | exon_MSTRG.351, exon_MSTRG.317, exon_MSTRG.218, exon_MSTRG.352 |
| GO:0043412 | macromolecule modification | Biological Process | GO:0043170 | macromolecule metabolic process | 0.6 | 1 | exon_MSTRG.218 |
| GO:0090304 | nucleic acid metabolic process | Biological Process | GO:0043170, GO:0006139 | macromolecule metabolic process, nucleobase-containing compound metabolic process | 0.36 | 1 | exon_MSTRG.317 |
| GO:0036211 | protein modification process | Biological Process | GO:0043412, GO:0019538 | macromolecule modification, protein metabolic process | 1 | 1 | exon_MSTRG.218 |
| GO:0016070 | RNA metabolic process | Biological Process | GO:0090304 | nucleic acid metabolic process | 0.6 | 1 | exon_MSTRG.317 |
| GO:0016071 | mRNA metabolic process | Biological Process | GO:0016070 | RNA metabolic process | 1 | 1 | exon_MSTRG.317 |

**Table S3 (b). List of molecular function gene ontologies (GO)**

| **GO ID** | **GO Name** | **GO Type** | **Parents (ACC)** | **Parents (Name)** | **Nodescore** | **#Seqs** | **Sequence Names** |
| --- | --- | --- | --- | --- | --- | --- | --- |
| GO:0003674 | molecular_function | Molecular Function |  |  | 1.8 | 4 | exon_MSTRG.351, exon_MSTRG.218, exon_MSTRG.317, exon_MSTRG.298 |
| GO:0005488 | binding | Molecular Function | GO:0003674 | molecular_function | 0.6 | 1 | exon_MSTRG.351 |
| GO:0003824 | catalytic activity | Molecular Function | GO:0003674 | molecular_function | 2.4 | 3 | exon_MSTRG.218, exon_MSTRG.317, exon_MSTRG.298 |
| GO:0016740 | transferase activity | Molecular Function | GO:0003824 | catalytic activity | 1 | 1 | exon_MSTRG.218 |
| GO:0016787 | hydrolase activity | Molecular Function | GO:0003824 | catalytic activity | 2 | 2 | exon_MSTRG.317, exon_MSTRG.298 |
| GO:0005515 | protein binding | Molecular Function | GO:0005488 | binding | 1 | 1 | exon_MSTRG.351 |
| GO:0140096 | catalytic activity, acting on a protein | Molecular Function | GO:0003824 | catalytic activity | 1 | 1 | exon_MSTRG.218 |

**Table S3 (c). List of cellular component gene ontologies (GO)**

| **GO ID** | **GO Name** | **GO Type** | **Parents (ACC)** | **Parents (Name)** | **Nodescore** | **#Seqs** | **Sequence Names** |
| --- | --- | --- | --- | --- | --- | --- | --- |
| GO:0005575 | cellular_component | Cellular Component |  |  | 0.43 | 2 | exon_MSTRG.160, exon_MSTRG.324 |
| GO:0110165 | cellular anatomical entity | Cellular Component | GO:0005575 | cellular_component | 0.72 | 2 | exon_MSTRG.160, exon_MSTRG.324 |
| GO:0031224 | intrinsic component of membrane | Cellular Component | GO:0110165, GO:0016020 | cellular anatomical entity, membrane | 1.2 | 2 | exon_MSTRG.160, exon_MSTRG.324 |
| GO:0016020 | membrane | Cellular Component | GO:0110165 | cellular anatomical entity | 0.72 | 2 | exon_MSTRG.160, exon_MSTRG.324 |
| GO:0016021 | integral component of membrane | Cellular Component | GO:0031224 | intrinsic component of membrane | 2 | 2 | exon_MSTRG.160, exon_MSTRG.324 |

**Table S4. List of primers used in RNA-Seq validation**

| **Primer name** | **Sequence 5’- 3’** | **Source** |
| --- | --- | --- |
| GAPDH_Fw2 | GCGTCAACCTTGAAGCCTAC | Yan et al., 2021 |
| GAPDH_Rv2 | TTACCAGAGGGACCGTCAAC |  |
| 18S rRNA_Fw | TGGAGGAAAACGGGCACTAC | Zheng et al., 2020 |
| 18S rRNA_Rv | GACTTGTCTGCGTTGCACAG |  |
| MSTRG.160_Fw | TAGATGTGGTGTGCAGCCAA | MSTRG.160 |
| MSTRG.160_Rv | GTAAGTGGGCAGCTTTCCGA |  |
| MSTRG.218_Fw2 | AGGCTTGCGTTTTCGACCT | MSTRG.218 |
| MSTRG.218_Rv2 | TTGATTCGCTGCCATACCCT |  |
| MSTRG.298_Fw2 | TCCTTCGGGTCAGTTTTCGG | MSTRG.298 |
| MSTRG.298_Rv2 | GTTGGACGATTTGCGACAGG |  |
| MSTRG.317_Fw2 | TCATCGCGTCTCGTACCAAG | MSTRG.317 |
| MSTRG.317_Rv2 | CGGCTGGATTATCGGTGGAT |  |
| MSTRG.324_Fw2 | GCTCGCAGTCGGATTTACCT | MSTRG.324 |
| MSTRG.324_Rv2 | GAGAAGCTTGCAAGGGTTGG |  |
| MSTRG.351_Fw | CCAGCGGCCATATTGAAACG | MSTRG.351 |
| MSTRG.351_Rv | TTTTCCAGCGGGTGTCATCA |  |
| MSTRG.352_Fw2 | AGAGACAGGCTGTAGACGCT | MSTRG.352 |
| MSTRG.352_Rv2 | GCATGGTGGCCAATAGAGGA |  |
